# Will the Establishment of a National Park Protect More Suitable Habitats for the Qinling Golden Snub‐Nosed Monkey?

**DOI:** 10.1002/ece3.72421

**Published:** 2025-11-02

**Authors:** Tong Wu, Xiaoxiao Shu, Xiaowei Wang, Haitao Zhao, Li Zhao, Shuaibin Shang, Yan Wang, Wei Li, Yi Ren, Weiwei Fu, Shujun He, Daibo Zhu, Bin Guo, Guiyuan Zhang, Chengliang Wang

**Affiliations:** ^1^ Shaanxi Provincial Field Observation and Research Station for Golden Monkey, Giant Panda and Biodiversity Shaanxi Institute of Zoology Xi'an Shaanxi China; ^2^ Key Laboratory of Qinling Medicinal and Edible Biological Resources Conservation and Utilization, College of Biology, Food and Chemistry Shaanxi Xueqian Normal University Xi'an Shaanxi China; ^3^ Shaanxi Key Laboratory for Animal Conservation, College of Life Sciences Northwest University Xi'an Shaanxi China; ^4^ Northwest Institute of Investigation, Planning & Design of the State Forestry & Grassland Administ Administration Xi'an Shaanxi China; ^5^ Management Bureau of Zhouzhi National Nature Reserve Xi'an Shaanxi China; ^6^ Xiaolongshan National Nature Reserve Management Center Tianshui Gansu China

**Keywords:** ecological corridors, habitat, Qinling mountains, Qinling National Park, *Rhinopithecus roxellana qinlingensis*

## Abstract

The Qinling Mountains, recognized as a biodiversity hotspot, are included in the national park construction plan, necessitating clear boundaries and critical habitats for endangered species, which are essential to fulfill the function of the national park. In this study, we used the MaxEnt model to analyze the suitable distribution area of the flagship species, the Qinling golden snub‐nosed monkeys ( *Rhinopithecus roxellana qinlingensis* ), and to identify conservation gaps for this species in the national park. The annual range area of the Qinling golden snub‐nosed monkey was set as the minimum threshold for high‐suitability habitat patches. We identified 30 high‐suitability habitat patches (8664 km^2^), with 1350 km^2^ located outside the national park. Additionally, we identified 58 ecological corridors, which included 44 ecological pinch‐points and 88 ecological barrier patches. Approximately 16% of the corridors and 29% of the ecological pinch‐points were located outside the national park. The primary barriers to these corridors were roads and buildings, corresponding to the key factors affecting the distribution of the Qinling golden snub‐nosed monkeys in the Qinling Mountains (human footprint). Projections for the year 2030 indicate that 9.89% (857 km^2^) of high‐suitability habitat patches would be affected under the natural development scenario (2020–2030 trend). However, in the national park scenario, a loss of 159 km^2^ of high‐quality habitat—projected under natural development—would be prevented. The national park will overcome the shortcomings of the decentralized protected area system, and future efforts may involve expanding boundaries or creating protection districts, alongside enhancing monitoring surveys and the construction of artificial corridors.

## Graphical abstract

The protection gap of high‐suitability habitat patches in national parks has decreased by 36.94%, compared with nature reserves. The loss of high‐suitability habitat patches in natural development scenario, with decreased 181 km^2^ in national park scenario by 2030.

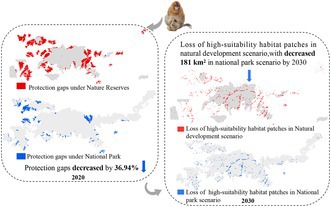

## 1 Introduction

The *Living Planet Report 2022* highlights that since 1970, the average relative abundance of monitored wild animal populations has declined by 69% globally (Almond et al. 2022). The *Kunming‐Montreal Global Biodiversity Goals* also emphasize the need to take proactive management actions to achieve the recovery and protection of species, especially those threatened (Dunn et al. 2023). As one of the earliest signatories and ratifiers of the *Convention on Biological Diversity*, the Chinese government places great emphasis on biodiversity conservation (Xu et al. 1999; Xu et al. 2012). The establishment of nature reserves is a key political strategy for protecting important wildlife (Shafer 1999; Geldmann et al. 2013). Currently, over 2700 different types of nature reserves have been established in China for preserving species diversity and ecosystem health (Wang et al. 2020; Allan et al. 2022; Zhao et al. 2024). However, there are still deficiencies in the design and management implementation of these nature reserves, leading to suboptimal protection outcomes (Leverington et al. 2010). Therefore, in 2013, the Chinese government formally proposed the establishment of national parks for the first time and subsequently launched 10 pilot parks, 5 of which have been completed and opened to the public (Zhao 2022). The establishment of the national park system integrates various types of nature reserves, offering higher ecological value, broader protection coverage, more intact ecosystems, and higher levels of management. This initiative addresses the shortcomings of the fragmented reserve system, achieving complete protection, systematic restoration, and unified management of one or more natural ecosystems (Liu 2023).

In September 2022, the National Forestry and Grassland Administration, the Ministry of Finance, the Ministry of Natural Resources, and the Ministry of Ecology and Environment jointly released the *National Park Spatial Layout Plan* (Tang 2020). The Qinling Mountains, renowned for their exceptional natural resources and pivotal ecological significance, were chosen as part of the second batch of approved national parks (Liu 2023). The Qinling Mountains, spanning central China, serve as a critical climatic boundary between northern and southern regions (Zhang et al. 2017). As a major zoogeographic divide between the Palearctic and Oriental realms (Holt et al. 2013), they represent an evolutionary ecotone that promotes species divergence and endemism (Shahzad et al. 2020; Liu et al. 2023). The region supports more than 560 terrestrial vertebrate species, accounting for nearly one‐fifth of China's total fauna (Liu et al. 2012). This includes over 50 endangered animal species, such as the giant panda ( *Ailuropoda melanoleuca* ) (Zhang et al. 2019). This exceptional biodiversity reinforces the Qinling Mountains as one of the most important biodiversity hotspots in China (Zhang et al. 2017). At present, the Qinling Mountains contain 33 nature reserves, covering a combined area of 5667 km^2^ (Liu 2023). However, due to administrative fragmentation and the small size of individual reserves, ecosystems have been artificially divided, reducing the effectiveness of habitat protection for endangered species (Li, Yu, et al. 2023). National parks, which are established at the national level, follow principles of ecosystem integrity, authenticity, and large‐scale conservation. These parks are designed to maintain large‐scale ecological processes and intact ecosystems, thereby addressing important shortcomings in the current network of existing protected areas (Zang et al. 2022). The establishment area of the Qinling National Park is located in the core area of the Qinling Mountains, integrating 65 existing natural protected areas, including 26 nature reserves, 30 forest parks, three wetland parks, two geological parks, and four scenic spots (Liu 2023). Determining potential areas and delineating spatial boundaries are crucial measures for the construction and management of the national park (Liu and Yu 2020; Xue et al. 2023), which are of great significance for maintaining the integrity and continuity of ecosystems within the national park (Belote et al. 2017; Xu et al. 2017). Delineation of national park boundaries should fully consider biodiversity and flagship species protection, ecosystem integrity, and green development space (Peng et al. 2021; Wang, Zhong, et al. 2023). Various factors such as species genetic differentiation, flagship species distribution, and landscape continuity can serve as reference principles for the delineation of national park boundaries (Kim et al. 2021).

**FIGURE 1:**
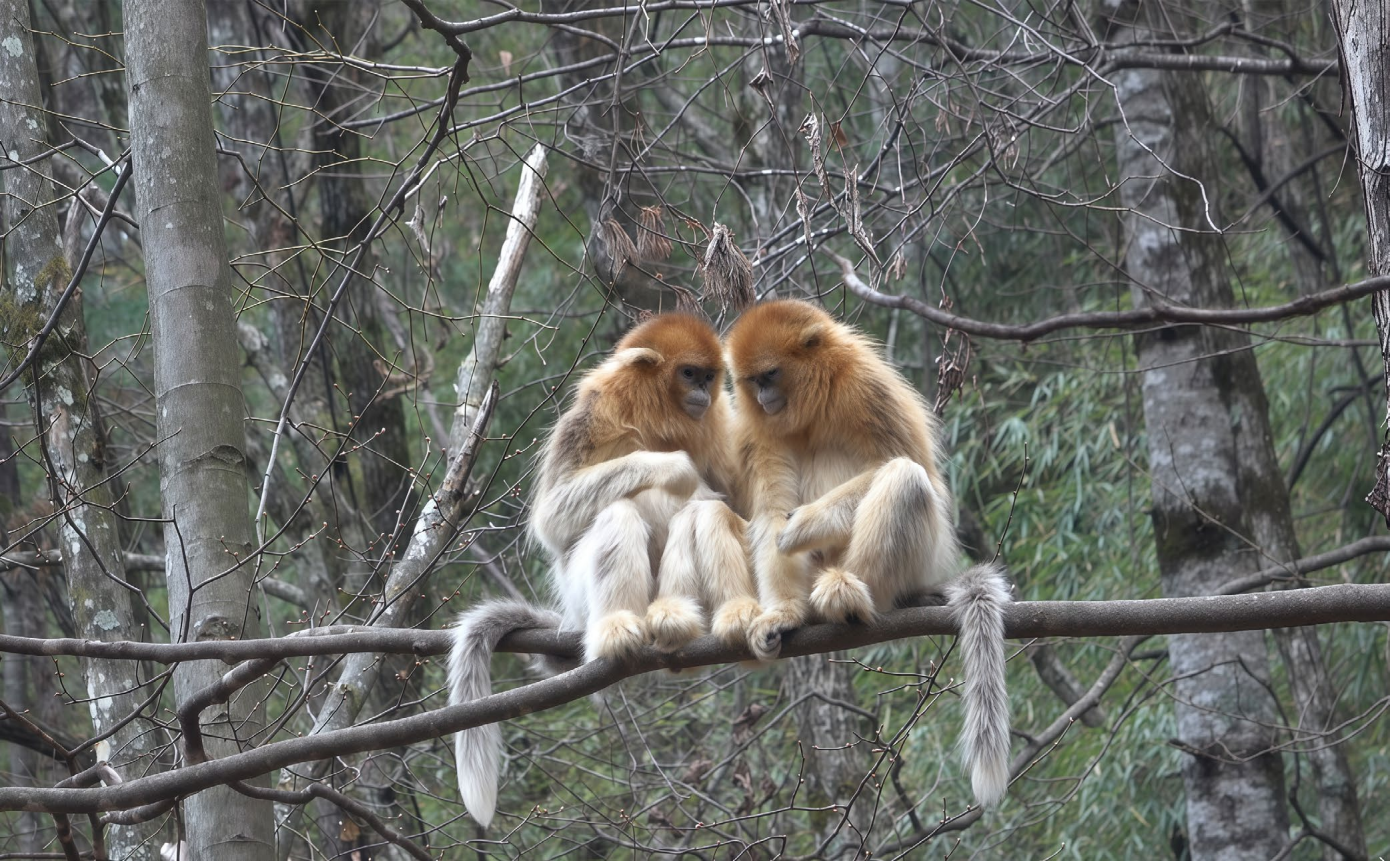
Qinling golden snub‐nosed monkey *Rhinopithecus roxellanae qinlingensis* (The photograph was taken in the Guanyinshan National Nature Reserve in November 2022).

The Qinling golden snub‐nosed monkey *Rhinopithecus roxellanae qinlingensis* (Figure 1), is a rare and endemic species of China and is categorized as a Class I protected animal in China. It is listed as an Endangered species on the IUCN Red List (IUCN 2015). The Qinling golden snub‐nosed monkeys primarily inhabit sparsely populated forest ecosystems of the Qinling mountains (Wang, Chen, et al. 2023). The Qinling golden snub‐nosed monkey serves as an important indicator species of natural ecosystems in the Qinling mountains. As a flagship species in China's ecological conservation, they rank second only to the giant panda in terms of conservation value (Yan et al. 2018). By safeguarding this species, we can provide an umbrella of protection for other animals, thus contributing significantly to the preservation of regional biodiversity (Johnson et al. 2017). However, the habitat of the species has undergone dynamic changes over the past four decades, and it currently confronts challenges such as gaps in habitat protection and maintaining connectivity. Consequently, protection strategies must be promptly adapted to address these evolving circumstances (Wang, Chen, et al. 2023). The construction of the Qinling National Park will integrate, optimize, and supplement multiple protected areas, playing a crucial role in addressing the conservation challenges faced by this species. Additionally, it will further enhance the umbrella protection role of this species and provide important references for the delineation of important potential areas and boundaries of the Qinling National Park.

This study focuses on the flagship species, the Qinling golden snub‐nosed monkey, in the Qinling region. Using ecological niche models and ecological corridor construction, the study aims to explore the primary ecological issues faced by this species regarding habitat and dispersal pathways. The objectives include (a) identifying important habitats and protection gaps for the Qinling golden snub‐nosed monkey; (b) identifying the distribution of key ecological corridors and pinch points; (c) clarifying the main factors influencing the future migration and dispersal of the Qinling golden snub‐nosed monkey; and (d) proposing suggestions for potential functional zones and boundary delineation for the construction of the Qinling National Park. The conclusions of this study not only contribute to improving and refining conservation and management strategies for the Qinling golden snub‐nosed monkey, addressing its ecological challenges, but also leverage its umbrella protection role, providing scientific foundations for the construction and management of the Qinling National Park.

## 2 Methods

### 2.1 Regional Overview

**FIGURE 2:**
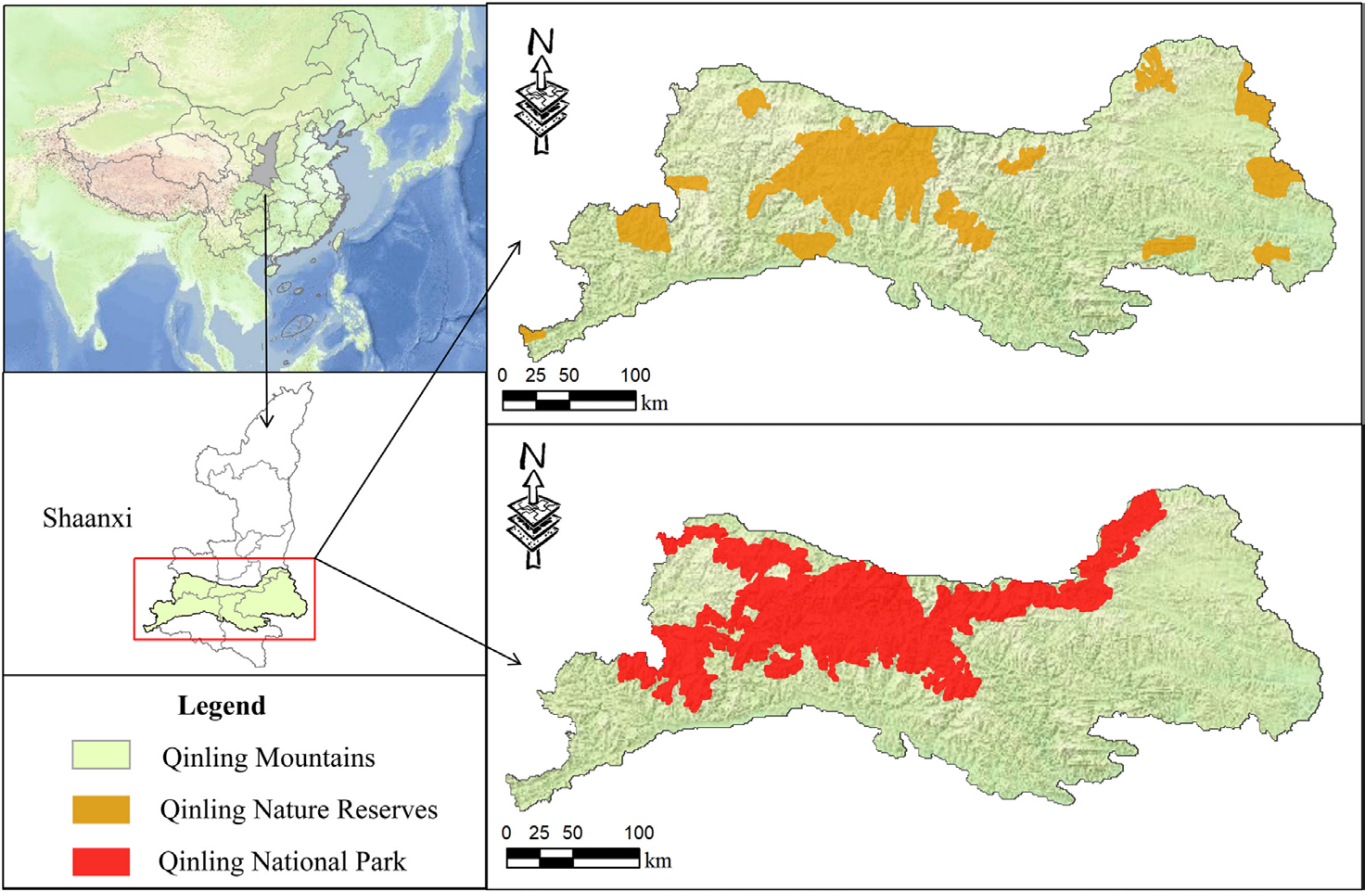
Location of the nature reserves and national park in Qinling Mountains, Shaanxi Province, China.

The Qinling Mountains serve as the research area of this study, located between 105°29′18″ E and 111°01′54″ E and 32°28′53″ N and 34°32′23″ N (Figure 2). It encompasses 39 counties and districts spanning Xi'an, Baoji, Weinan, Hanzhong, Ankang, and Shangluo in the Shaanxi Province, with a total area of 58,200 km^2^. The northern part of the Qinling Mountains belongs to the Yellow River basin, characterized by steep terrain and a warm temperate semi‐humid monsoon climate. The southern part belongs to the Yangtze River basin, featuring gentle slopes, widespread hills, and a humid subtropical monsoon climate. The mountains boast an average elevation exceeding 1000 m, culminating in a peak that reaches a towering height of 3771 m. With an annual average precipitation of 820 mm, the Qinling Mountains play a pivotal role in water conservation in China (Bian et al. 2016; Soo et al. 2022). Furthermore, the Qinling Mountains serve as a sanctuary for a rich biodiversity, having provided refuge during the Quaternary glacial period (Wang et al. 2013). This has allowed for the preservation of rare and endangered species such as giant pandas, golden snub‐nosed monkeys, and takins ( *Budorcas taxicolor* ), which have flourished here, forming unique geographic populations. The total area of Qinling National Park is 12,900 km^2^, ranging from 33°16′06″ N to 34°32′19″ N and from 106°16′24″ E to 110°11′43″ E (Figure 2). It involves 21 counties (cities, districts) and 106 townships (towns) across six cities in Shaanxi Province, including Xi'an, Baoji, Weinan, Hanzhong, Ankang, and Shangluo (Liu 2023).

### 2.2 Data Collection

From 2019 to 2021, historical and current distribution areas of golden snub‐nosed monkeys in the Qinling Mountains within Shaanxi Province and field visits to 32 nature reserves in the Qinling region were conducted. The investigation included transect analysis, infrared camera monitoring techniques, and infrared thermal imaging drone survey techniques. Simultaneously, field survey data during 2020–2021 from nature reserves included information on species occurrence location, population size and structure, habitat characteristics, survival status, migration route, historical range dynamics, and conservation status. In parallel, a comprehensive review was conducted of literature, books, monographs, survey reports, and other relevant sources pertaining to golden monkey surveys in the Qinling region published between 2019 and 2021. Analyzing and summarizing the recorded information on Qinling golden snub‐nosed monkeys, stable locations, and occurrence information of their family groups were obtained. The distribution points of golden snub‐nosed monkeys were selected using resolution distance to reduce spatial autocorrelation of samples caused by duplicate species distribution points in the same geographical grid (Chi et al. 2019). This study used ArcGIS 10.7 to set the resolution distance to 1 km. After screening, a total of 116 distribution points of golden snub‐nosed monkey family groups were obtained for model evaluation.

### 2.3 Habitat Variable Processing

The evaluation of variables affecting the distribution of golden snub‐nosed monkeys in the Qinling Mountains is divided into three categories. (1) Six geographic environmental variables, including elevation, slope, aspect, distance to roads, distance to residential areas, and distance to water sources. We obtained digital elevation model (DEM) data from the Resource and Environmental Data Cloud Platform of the Institute of Geographic Sciences and Natural Resources Research, CAS (http://www.resdc.cn), from which we derived elevation, slope, and aspect data variables. Road and hydrological data were also obtained from this platform. Population data were downloaded from the National Catalog Service for Geographic Information (http://www.webmap.cn) to form the variable of residential areas in the research area. The Euclidean distance algorithm in ArcGIS 10.7 was used to obtain data variables for distance to roads, distance to residential areas, and distance to water sources. (2) Two vegetation variables, including normalized difference vegetation index (NDVI) and vegetation type. We obtained NDVI data from the Resource and Environmental Data Cloud Platform (http://www.resdc.cn) and vegetation distribution type data from the Geographic Remote Sensing Ecological Network Platform (http://www.gisrs.cn). (3) One human activity variable, i.e., human footprint data. This study obtained human footprint data from the Introduction for Global Human Footprint Dataset (version 2020) (Mu et al. 2022).

Using ArcGIS 10.7 software, all variable data were resampled at a resolution of 1 km and converted to “.asc” format. To avoid autocorrelation and multicollinearity among variable data, this study extracted attribute values of environmental variable data corresponding to the golden snub‐nosed monkey points. SPSS 25.0 was employed to calculate the Pearson correlation coefficient of the environmental variable attribute values for golden snub‐nosed monkeys. Environmental factors with correlation coefficients smaller than 0.80 were retained for model calculations (Johnson et al. 2016).

### 2.4 Identification of Important Habitats

To simulate the spatial distribution of species habitat suitability, the Maximum Entropy Model (MaxEnt) model, a type of ecological niche model, is widely used in wildlife habitat research due to its simplicity of operation and reliable predictive performance (Wang, Chen, et al. 2023; Jiang et al. 2024). In this study, the identification of important habitats for the golden snub‐nosed monkey was conducted using the MaxEnt model. R 4.2.2 was employed, and the model utilized the ‘kuenm’ package (Cobos et al. 2019). The training dataset consisted of 75% of the golden snub‐nosed monkey distribution points, while the remaining 25% were designated as the testing dataset. The ‘reg_mult’ parameter was set from 0.5 to 4.0 in increments of 0.5, and the ‘f_class’ was set to include 29 combinations. The evaluation criterion for the optimal model was set as delta_AICc = 0 and an omission rate below 2.0%.

After computation, the β values of the optimal model and model combinations were input into MaxEnt v3.4.4 for 50 iterations (Merow et al. 2013; Chi et al. 2019). This study used Receiver Operating Characteristic (ROC) curves and Area Under the Curve (AUC) values as metrics to assess the performance of the model. The AUC value, ranging from 0.5 to 1.0, serves as a critical criterion for evaluating the accuracy of the MaxEnt model. Notably, a higher AUC value indicates a closer alignment between the model's predictions and the actual spatial distribution of wildlife (Jiang et al. 2024).

The generated habitat layers were classified into four categories based on the probability values of suitability zones. The suitability values have been categorized as follows: 0–0.098 represents unsuitable areas, 0.098–0.270 signifies low suitability areas, 0.270–0.477 corresponds to medium suitability areas, and 0.477–0.830 denotes high suitability areas. Notably, the high suitability areas have been designated as crucial habitats for the golden snub‐nosed monkeys. Long‐term ecological studies in the Qinling region have demonstrated that the annual home range size of golden snub‐nosed monkeys is between 18.29 and 22.5 km^2^ (Li et al. 2001; Guo 2004). This study referenced these findings and set the minimum threshold for the optimal habitat patch area to be 23.00 km^2^ for Qinling golden snub‐nosed monkeys. High suitability patches that do not fall entirely within the boundaries of the national park are defined as protection gap patches.

### 2.5 Prediction of Important Factors for Habitat Suitability

To analyze temporal changes in human impact, we aggregated human footprint index (HFI) data from 2000 and 2020 to construct a space–time cube with an annual time step using the Space–Time Pattern Mining tool in ArcGIS Pro 3.0.2. (Table S3). Subsequently, we employed the curve fit forecasting method to generate a predictive layer for 2030, which was defined as the natural development scenario (Table S4).

### 2.6 Effectiveness of National Park Construction

To evaluate future conservation outcomes, this study simulates the 2030 national park scenario by extrapolating the projected management effectiveness of current nature reserves. Our modeling assumes equivalent management effectiveness between future national parks and existing reserves in 2030.

We quantified the Management Effectiveness Indicator as the temporal difference in the Human Footprint Index within nature reserves between 2020 and 2030. The 2030 Human Footprint Index values were calculated using the methodology outlined in Section 2.5.

The construction of the national park will encompass currently unprotected zones (designated as “new areas”). For these regions, the 2030 Human Footprint Index was calculated by adding the baseline 2020 Human Footprint Index to the projected Management Effectiveness Indicator of the national park.

The vector map of nature reserves was divided into 1 × 1 km grid cells, which were then converted into a point layer. Habitat factor data for each point were extracted from the habitat variable layers in Section 2.3, along with additional variables, including population and regional GDP for the year 2020. The data source was the Resource and Environmental Data Cloud Platform (http://www.resdc.cn).

The difference in the Human Footprint Index (HFI) between 2030 and 2020 within the nature reserve was used as the dependent variable, while the habitat factors served as independent variables in constructing the model. Based on the results of the normality test, it was found that both the HFI difference and the habitat variables did not follow a normal distribution. Therefore, non‐normal models were employed to analyze the relationship between the HFI difference and the habitat variables. Specifically, the ‘mgcv’ package and ‘xgboost’ package were used to develop GAM and XGBoost models separately (Chen and Guestrin 2016; Wood et al. 2016). Additionally, the GAM and XGBoost models were integrated into an ensemble model to enhance the predictive power. To evaluate the performance of the models, the Mean Squared Error (MSE) was calculated for each of the three models. The MSE values were 14.77 for the GAM model, 15.37 for the XGBoost model, and 14.68 for the ensemble model. Based on these results, the ensemble model was selected as the optimal model.

To evaluate the management effectiveness of the national park in the new areas, quantified by the HFI difference (2030–2020), we applied the optimal model using the habitat variables from these areas. First, the 2020 HFI values from the new areas were combined with the projected changes to generate the 2030 HFI layer. Subsequently, the 2030 HFI layers of both new and existing nature reserves were merged to create a unified HFI layer representing the 2030 national park scenario.

We integrated the 2030 human footprint layers of both scenarios into MaxEnt v3.4.4, conducting 50 iterations to project the species' suitable distribution areas in 2030. The methodology and parameters utilized in this process followed those described in Section 2.4.

### 2.7 Construction of Ecological Corridors

The ecological corridors were constructed using the circuit theory model Circuitscape 4.0.5 (McRae et al. 2013). In ArcGIS 10.7, the Pinchpoint Mapper tool from the Linkage Mapper 2.0.0 toolkit was employed for this purpose (McRae 2012). The source sites for ecological corridors were identified as the crucial habitat patches of the Qinling golden snub‐nosed monkeys. An ecological resistance layer was then generated by subtracting the habitat suitability layer from 1. The Conefor 2.6 software package was used to calculate the landscape connectivity between different patches (Saura and Rubio 2010), with a Cost‐weighted distance threshold of 200,000 set for truncating corridors. Ecological pinch points are critical points with high flow within the ecological corridor, displaying irreplaceability in connectivity, and should be prioritized for protection. Analysis of pinch points in ecological corridors was conducted using the Pinchpoint Mapper tool, with a CWD cutoff distance (cost‐weighted corridor ‘width’) set to 100,000. Circuitscape mode for raster centrality calculations was selected as ‘All to one’. Ecological barriers refer to the areas within the corridors where animal movement is impeded, and removing these barriers can increase corridor connectivity. Barrier analysis was performed using the Barrier Mapper tool, with a search radius set to 1 km, and the method for combining across multiple core area pairs was set as ‘Maximum’. Layers of ecological pinch points and barriers were classified into four categories based on an exponential gradient, with the highest numerical value determining the critical pinch point areas and barrier areas (Wang et al. 2024). The index range for ecological pinch points is 0 ~ 49, and 25 ~ 49 is selected as the pinch point area for analysis. The index range for ecological barriers is 0 ~ 98, and the area with an index of 66 ~ 98 is selected as the barrier area. The alteration in land use type exhibits a strong correlation with the human footprint index (Di Marco et al. 2018; Williams et al. 2020). Environmental variable values were extracted from pinch points and barrier areas, referencing satellite images (July 2021, resolution: 15 m, data source: https://www.tianditu.gov.cn/). The main variables affecting pinch points and barrier areas are analyzed based on the percentage of each variable to the total variable as a measure of the multiplicity of that variable.

## 3 Results

### 3.1 Habitat Suitability and Important Habitat

The correlation coefficients of the nine variables in the three categories were all calculated to be less than 0.80 (Table S1). Therefore, all nine variables with low correlations were used to construct the MaxEnt model. After computation, the value of β was 2.0, and the delta AIcc value of the model was 0, with an Omission rate of 0.03 < 2.00, indicating the optimal model. The AUC values of the training set were above 0.900 in all MaxEnt models, suggesting an excellent fit of the models, indicating a high reliability in predicting suitable habitats.

Model analysis revealed that elevation and human footprint were the two variables contributing the most to the model, each accounting for over 40%. The other seven environmental variables—NDVI, distance to residential areas, distance to roads, land use type, distance to water sources, aspect, and slope—contributed less to the model, with contributions all below 6% (Table S2).

The unsuitable area for the Qinling golden monkey habitat covers 42,622 km^2^, the medium‐suitability area spans 10,193 km^2^, and the high‐suitability area, which encompasses 9210 km^2^, accounts for 11.76% of the Qinling Mountains.

**FIGURE 3:**
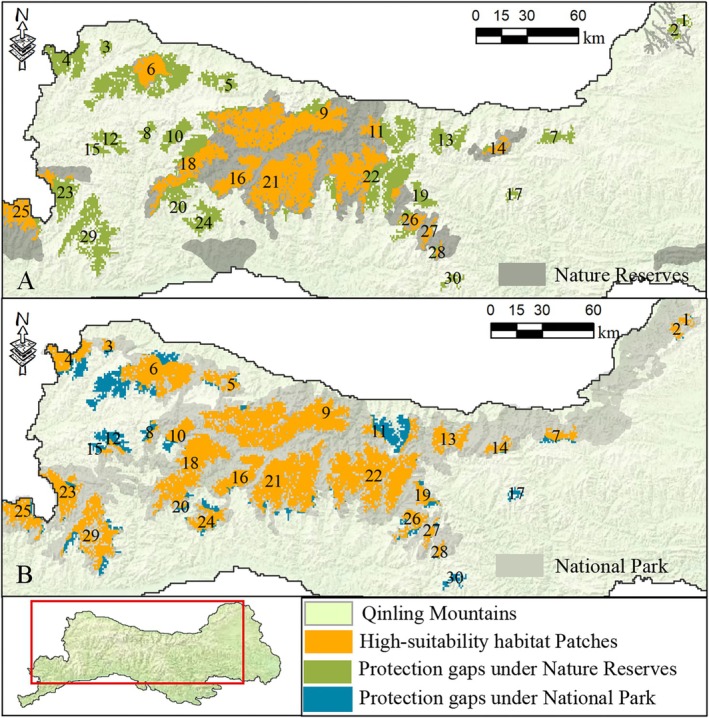
Location of high‐suitability habitats and protection gaps for the Qinling golden snub‐nosed monkey in Nature reserves and National parks. The numbers from 1 to 30 represent individual patches of high‐suitability habitat for this species.

The high‐suitability habitat areas form patches distributed throughout the Qinling Mountains, with a total of 30 patches covering an area of over 23 km^2^, totaling 8664 km^2^, which accounts for 94.05% of the total area of high‐suitability habitat for the Qinling golden snub‐nosed monkeys (Figure 3). Among these, 15 high‐suitability habitat patches are located within nature reserves, covering only 47.48% of the total area of high‐suitability habitat patches. However, the national park contains parts of 28 high‐suitability habitat patches, with 84.42% of high‐suitability habitat patches located within the national park. The proportion of high‐suitability habitat areas for the golden snub‐nosed monkey protected within the national park has significantly increased.

However, it is worth noting that only one high‐suitability habitat patch, namely No. 13, is entirely contained within the national park. Conversely, parts of 29 patches extend beyond the national park area, of which patches No. 4, No. 6, No. 11, and No. 12 have areas exceeding 100 km^2^ outside the national park. Additionally, highly suitable habitat patches No. 17 and No. 30 are both located outside the current nature reserves and national park, as illustrated in Figure 3.

The main influencing factors of high‐suitability habitat are elevation and human footprint, contributing over 40%, respectively (Table S1). The most suitable elevation range is between 1100 and 3200 m, with a human footprint index smaller than 5.32.

**FIGURE 4:**
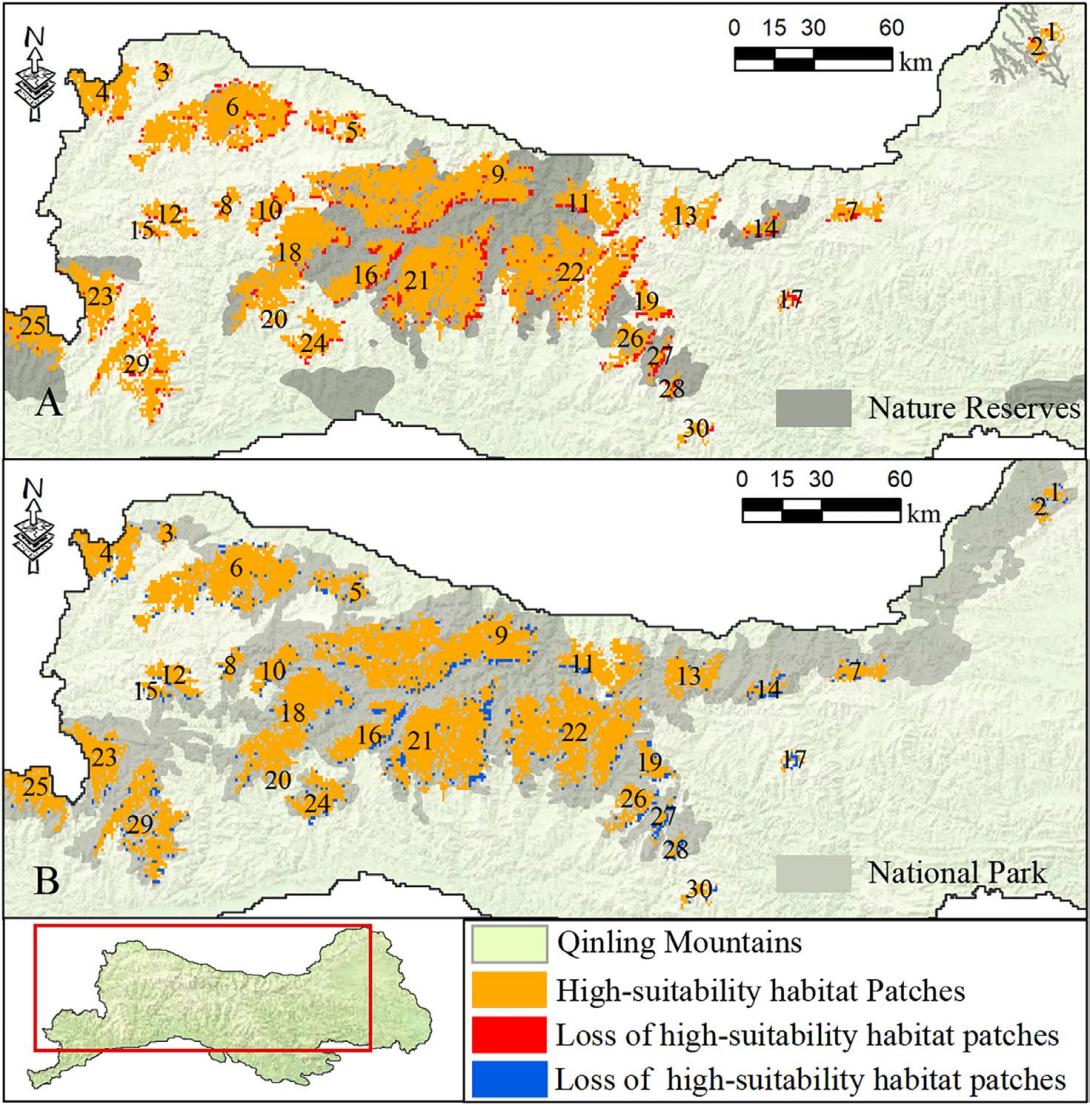
Changes in high‐suitability habitat patches from 2020 to 2030 under two scenarios: (A) natural development scenario and (B) national park scenario.

Under the natural development scenario, the high‐suitability habitat patches are currently experiencing significant impacts and threats. As illustrated in Figure 4, a decline in the habitat suitability index is projected by 2030, affecting 9.89% (857 km^2^) of these patches. The suitability habitat index of 30 patches is expected to decrease to varying degrees, with patches No. 17, No. 27, and No. 28 showing significant declines, impacting approximately 50% of their respective areas. In addition, the suitability habitat index for areas exceeding 100 km^2^ is anticipated to decrease in patches No. 9. Notably, 58.10% (498 km^2^) of the areas experiencing decreased suitability are located outside the national reserves.

Under the national park scenario, highly suitable habitat patches face potential contraction pressure. Notably, this represents a substantial reduction compared to the natural development scenario, particularly in preserving high‐quality habitats in the “new areas” designated for the national park. Additionally, the conservation measures are projected to prevent the loss of 181 km^2^ of high‐quality habitat that would otherwise occur under the natural development scenario. Importantly, 83.42% (151 km^2^) of these preserved habitats are located within the national park boundaries.

### 3.2 Qinling Golden Snub‐Nosed Monkey Corridors and Ecological Pinch Points

**FIGURE 5:**
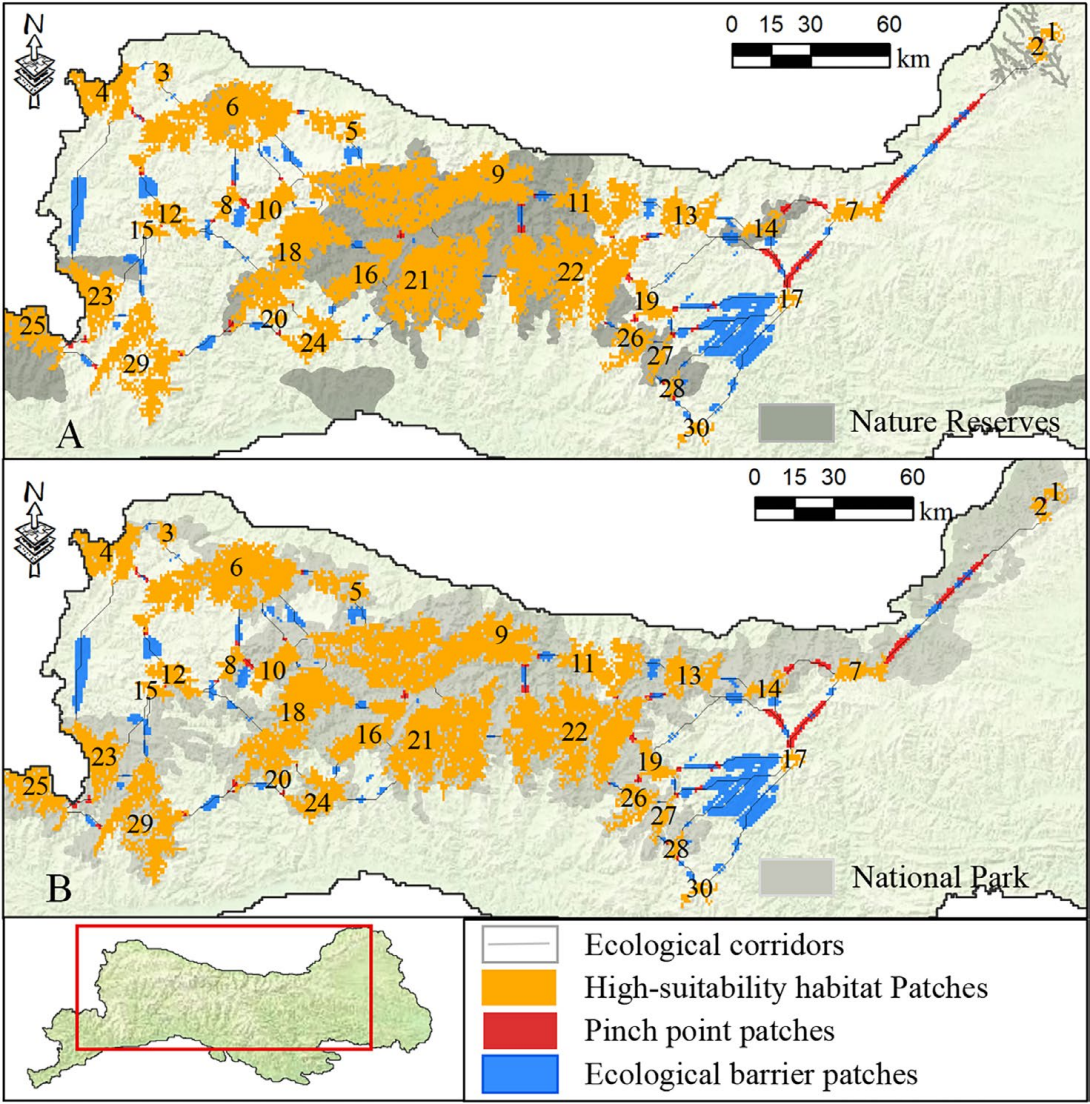
Ecological corridors, pinch point patches and ecological barrier patches between the high‐suitability habitat patches (A: Qinling nature reserves, B: Qinling national park).

This study identified 58 ecological corridors for the Qinling golden snub‐nosed monkey's important habitats using the minimum cost distance method based on circuit theory. The lengths of these corridors range from 1.41 km to 82.54 km. Among them, 27 corridors partially overlap with nature reserves, while 31 corridors are entirely outside the nature reserves. 48 corridors are partially within the national park, with particular attention needed for the 9 corridors entirely outside the current nature reserves and future national park boundaries (Figure 5).

Within this range, there are 44 pinch point patches, with areas ranging from 1 to 58 km^2^, 13 of these pinch point patches partially overlap with nature reserves, while 31 are located outside nature reserves. The national park boundaries contain 31 pinch point patches, while only 13 are outside this area (Figure 5).

Within this range, there are 81 ecological barrier patches, with patch areas ranging from 1 to 486 km^2^. The national park contains 40 patches, with 41 barrier patches located outside the park boundaries. Compared to the national park, 67 barrier patches are located outside nature reserves, while only 14 barrier patches are within nature reserves (Figure 5).

The primary threat factors to ecological pinch points and ecological barrier patches within nature reserves and the national park mainly arise from changes in land use types, such as roads, villages, farmland, and multiple disturbances caused by human activities such as gathering herbs, grazing, and tourism (Table 1). Unlike ecological pinch point patches, barrier patches have a higher proportion of road disturbance factors, reaching over 40% both inside protected areas and the national park, indicating that road factors may become one of the main disturbance threats to the migration of Qinling golden snub‐nosed monkeys in the future (Table 1).

**TABLE 1:** Major influencing factors of ecological pinch points and barrier patches.

Factors	Ecological pinch points (%)				Ecological barrier zones (%)			
	Nature reserves		National park		Nature reserves		National park	
	Inside	Outside	Inside	Outside	Inside	Outside	Inside	Outside
1. Road	11.76	34.48	29.17	29.63	48.57	37.43	42.31	36.44
2. Town	0.00	1.72	0.00	3.70	0.00	9.63	7.69	8.47
3. Residential areas	17.65	15.52	16.67	14.81	22.86	19.79	18.27	22.03
4. Farmland	35.29	36.21	33.33	40.74	22.86	31.02	26.92	32.20
5. Human activities	35.29	8.62	20.83	3.70	0.00	1.60	1.92	0.85
6. Waters	0.00	3.45	0.00	7.41	2.86	0.53	1.92	0.00
7. Mining	0.00	0.00	0.00	0.00	2.86	0.00	0.96	0.00

## 4 Discussion

### 4.1 Gap in Golden Snub‐Nosed Monkey Protection

Highly suitable habitat patches are the foundational spaces for the survival of endangered species (Wang et al. 2024). Currently, there is no uniform standard for delineating the most suitable habitat patches (Wegge et al. 2018; Shanu et al. 2019). For instance, in studies on *Panthera tigris altaica* , the minimum home range has been used as the criterion for the most suitable habitat patches (Jin 2023). In our study, the maximum home range throughout the year was used as the criterion, considering the wide‐ranging activities of the golden snub‐nosed monkeys and their varying seasonal activity areas in migration patterns (Fan et al. 2019). This approach maximizes the preservation of intact habitat patches, avoiding habitat fragmentation and the impacts of small patches on species conservation. For endangered species such as the golden snub‐nosed monkey, highly suitable habitat patches can serve as foundational conservation units, providing greater spatial precision than traditional range‐based occurrence mapping (Yu et al. 2022).

In comparison to the existing nature reserve boundaries, over 50% of the highly suitable habitat patches are located outside these areas, lacking effective maintenance and supervision. While the national park encompasses most of the highly suitable patches, 15.58% of the regions remain unattended, mainly concentrated in the northwest and central directions of the national park. Based on the degree of completeness and high‐level vacancy, 11 priority protection gap patches are identified. Among them, Patch 6 and Patch 8 have had the distribution of species effectively validated in two news articles. The news reports indicate that in January 2021 and March 2021, the first sightings of golden snub‐nosed monkey activity were recorded respectively in Dongheqiao Village, Huangniupu Town, Fengxian County (western part of Patch No. 6) (https://www.thecover.cn/news/6729066), and Daotiejin Village, Pingkan Town, Fengxian County (Patch No. 8) (https://www.sohu.com/a/454910730_100185418), both outside the boundaries of the national park. Witness evidence has confirmed the reliability of the identification of the most suitable distribution areas in this study and underscores the need for relevant authorities to strengthen species monitoring and investigation in the protection gap areas.

### 4.2 Main Environmental Variables Influencing the Distribution of Qinling Golden Snub‐Nosed Monkey

In recent years, the Qinling Mountains have benefited from conservation projects such as natural forest protection and ecological migration, resulting in forest restoration. Currently, the vegetation coverage has exceeded 84% (Zhao et al. 2019). Vegetation‐related environmental factors have a minor influence on the Qinling golden snub‐nosed monkey, while elevation (45.7%) and human footprint (40.6%) emerge as the main environmental variables affecting the distribution of the species. Elevation significantly impacts the microclimate, ecosystem composition, and species composition in the Qinling Mountains (Yu et al. 2013; Zhang et al. 2019). Human activity, on the other hand, is a major threat to the survival and distribution of most wildlife species as well as global biodiversity (Wang et al. 2024). As altitude increases, human settlements typically decrease, and the human footprint index generally declines. However, certain high‐altitude areas may serve as tourist destinations or sites for herb collection (Wang, Zhao, et al. 2023). These activities likely contribute to the lack of a significant correlation between altitude and the human footprint index in the Qinling region (Jiang 2019). This study predicts the average elevation range for the most suitable distribution of the Qinling golden snub‐nosed monkey to be between 1100 m and 3200 m, surpassing the highest elevation of actual distribution (2480 m) (Wang, Chen, et al. 2023). The elevations of 1500 m and above receive strict protection as key and core protected areas in the Qinling Mountains (Qinling Ecological Environment Protection Committee of Shaanxi Province 2019). Areas below the elevation of 1500 m experience more frequent human activities. Due to their shy nature, golden snub‐nosed monkeys typically avoid areas with human activity (Huang et al. 2021). However, recent surveys have found isolated activities of golden snub‐nosed monkeys at lower elevations (around 1000 m), possibly linked to human relocation and the implementation of returning farmland to forests policies (Wang, Chen, et al. 2023). Decreased human activity indices facilitate the expansion of golden snub‐nosed monkeys to lower elevations.

Among the main influencing factors, the human footprint is showing a continuous upward trend over the next 10 years (by 2030). The human footprint index in the marginal areas of high‐suitability habitat patches will continue to strengthen, leading to the encroachment of animals' high‐suitability habitats (Bodo et al. 2021). In recent years, national and local governments have been consistently strengthening the economic development and infrastructure construction in the Qinling‐Bashan Mountain areas (Shaanxi Provincial People's Government 2021). The expansion of urban and rural areas and outdoor tourism activities resulting from these projects will continue to increase, posing a threat to the future survival of the Qinling golden snub‐nosed monkey (da Silva et al. 2018). Areas outside the Qinling National Park, due to their lower land protection levels, will face increased risks from engineering projects (Alberts et al. 2021). These factors urge for rational planning of the boundary scope during the construction of the national park. Additionally, leveraging the ongoing implementation of policies for the protection of important wildlife habitats is crucial (National Forestry and Grassland Administration 2023). This involves establishing important habitats and elevating the level of land protection for habitats, aiming to better preserve intact and high‐suitability habitat patches.

### 4.3 Ecological Corridors and Key Points for Qinling Golden Snub‐Nosed Monkey

Ecological corridors are vital pathways for wildlife migration and dispersal (Wang and Li 2021). They play a pivotal role in maintaining the connectivity of isolated habitat patches, which is vital for mitigating population isolation and genetic decay caused by habitat fragmentation. This is essential for sustaining appropriate population sizes, preserving genetic diversity, and enhancing ecosystem potential (Ding et al. 2023). Furthermore, ecological pinch points and barrier patches are crucial nodes that determine the long‐term functionality of ecological corridors and represent areas where species conservation management should be prioritized (Wang et al. 2024). A total of 58 ecological corridors and 44 ecological pinch points have been identified, with over 50% of them located outside nature reserves. The establishment of a national park will help maintain more ecological corridors and pinch points, reducing the protection gaps for corridors to 16% and pinch points to 29%. However, there are still nine corridors and 13 ecological pinch points, concentrated in the northwestern and central regions, that remain inadequately protected. Nevertheless, the connectivity of 16 highly suitable patches will be impacted, particularly patches No. 6 and No. 17, which have several ecological corridors and pinch points located outside the national park. These areas require emphasis from relevant departments to strengthen corridor construction and maintain ecological pinch points. Additionally, the ecological pinch points between patches No. 2, No. 7, and No. 14 within the national park are significant in size and require proper management and maintenance.

Ecological barrier patches pose threats that must be eliminated from ecological corridors, necessitating the implementation of practical strategies to restore or enhance connectivity (Li, Liu, et al. 2023). This study revealed that the primary barriers to the dispersal corridor are roads and urban buildings. The obstruction of biological dispersal due to human road construction has significantly increased the risk of wildlife roadkill (Collinson et al. 2019; Hetman et al. 2019). Indeed, a road‐kill incident involving a Qinling golden snub‐nosed monkey was documented on the Zhuque section of the Xi'an‐Hanzhong highway in 2023, which lies between high‐suitability habitat patches No. 9 and No. 11 (personal communication). Going forward, field surveys of barrier patches within national park corridors should be intensified. It is crucial to investigate the specific types of obstructions between patches No. 6 and No. 8, as well as between patches No. 5 and No. 9, and to explore potential improvements through the construction of artificial corridors, such as tunnels or bridges.

### 4.4 Conservation Implications

The identification of high‐suitability habitat patches and the determination of important corridors and key patches for the golden monkeys in the Qinling Mountains serve as crucial references for the construction and management of Qinling National Park. This endeavor is of great significance in promoting the development of national ecological civilization. Based on these findings, several recommendations for construction and protection management can be proposed: (1) Strengthen Monitoring and Maintenance: Enhance monitoring of the 16 protection gaps and maintain the ecological pinch points. For high‐suitability habitat patches that are continuously distributed over large areas, consider appropriately expanding the boundaries of the national park to effectively manage and preserve these suitable habitats. In cases of sporadic and smaller suitable areas, implement salvage‐type protection by establishing new protected areas or designated protection plots. (2) Restore and Construct Ecological Corridors: The restoration and creation of ecological corridors facilitate wildlife migration and gene flow, playing a vital role in maintaining species diversity and enhancing the overall stability of the ecosystem (Peng et al. 2021; Li, Liu, et al. 2023). The under‐construction Qinling National Park should consider the installation of artificial corridors across highways and other infrastructure, including bridges and tunnels. In addition to utilizing eye‐catching signage to encourage drivers to remain vigilant for wildlife, this has proven to be an effective measure in many national parks (Shilling et al. 2020). Areas with low woodland quality and sporadic agricultural land within the Qinling Core Protection Area (elevation ≥ 1500 m) should be enhanced through afforestation and natural regeneration. These efforts aim to improve vegetation quality, reduce disturbance factors in ecological pinch points and barrier areas, and enhance habitat quality for animals. Additionally, promoting vegetation restoration along ecological corridors will help connect fragmented habitats and optimize conditions for golden snub‐nosed monkeys and other wildlife. (3) Enhance Boundary Monitoring: Establishing a robust national park boundary monitoring system is essential for ensuring the integrity and controllability of park boundaries. Measures should be implemented to prevent illegal activities, such as vandalism and hunting, as well as management strategies to mitigate human‐wildlife conflicts (Ndayishimiye et al. 2023).

## 5 Conclusion

In this study, we investigated the optimal distribution area and ecological corridor protection gaps for the Qinling snub‐nosed monkey based on its known distribution sites and the boundaries of Qinling National Park. Our findings indicate that the annual home range of the Qinling snub‐nosed monkey (23 km^2^) serves as the minimum threshold for identifying optimal habitat patches. We identified a total of 30 highly suitable habitat patches covering an area of 8664 km^2^, with 15.58% located outside the national park. Additionally, we identified 58 optimal ecological corridors, which include 44 ecological pinch points and 88 ecological obstacles. Notably, 16% of these corridors and 29% of pinch points were situated outside the protection area and have not been effectively safeguarded. Elevation and human activity are the primary factors influencing the distribution of the Qinling snub‐nosed monkey. It is projected that over 800 km^2^ of suitable habitat will face the risk of loss in 2030 due to increasing human encroachment. However, the establishment of the national park and a scientific conservation management system will significantly mitigate the loss of high‐suitability habitats. To address conservation gaps, we recommend expanding the national park boundaries or establishing additional protection zones based on the optimal distribution range of the Qinling snub‐nosed monkey. Strengthening monitoring surveys and constructing artificial corridors will also be vital. The identification of suitable habitats and ecological corridors for the Qinling snub‐nosed monkey, along with an analysis of the factors affecting them, is crucial for the effective conservation and management of this species. Moreover, these efforts will aid in delineating important functional zones and boundary lines for Qinling National Park.

## Author Contributions

**Tong Wu:** conceptualization (lead), methodology (lead), writing – original draft (lead). **Xiaoxiao Shu:** formal analysis (equal), writing – original draft (equal). **Xiaowei Wang:** conceptualization (equal), supervision (equal). **Haitao Zhao:** resources (equal), writing – review and editing (equal). **Li Zhao:** data curation (equal), writing – review and editing (equal). **Shuaibin Shang:** resources (equal). **Yan Wang:** data curation (equal). **Wei Li:** investigation (equal). **Yi Ren:** resources (equal), writing – review and editing (equal). **Weiwei Fu:** visualization (equal). **Shujun He:** project administration (equal). **Daibo Zhu:** project administration (equal). **Bin Guo:** data curation (equal). **Guiyuan Zhang:** resources (equal). **Chengliang Wang:** writing – review and editing (equal).

## Conflicts of Interest

The authors declare no conflicts of interest.

## Supporting information


**Table S1:** ece372421‐sup‐0001‐Tables.docx.

**Appendix S1:** ece372421‐sup‐0002‐AppendixS1.rar.

## Data Availability

All necessary data is available on the link: https://doi.org/10.6084/m9.figshare.30396997.v1.
