# Supplementary material for: Will the Establishment of a National Park Protect More Suitable Habitats for the Qinling Golden Snub‐Nosed Monkey?
Source: Ecol Evol. 2025 Nov 2;15(11):e72421. doi: 10.1002/ece3.72421 (PMC12579971; doi:10.1002/ece3.72421)
Supplement: Supplementary file 1 — Table S1: ece372421‐sup‐0001‐Tables.docx. [file ECE3-15-e72421-s002.docx]

**Supplementary Materials and Methods**

Table S1 Pearson correlation between environmental variable layers

| Layer | Aspect | elevation | human footprint index | landtype | distance to residential areas | ndvi | distance to roads | slope | distance to water sources |
| --- | --- | --- | --- | --- | --- | --- | --- | --- | --- |
| Aspect | 1 | 0.01 | -0.01 | 0.02 | 0.01 | -0.07 | -0.002 | 0.01 | -0.01 |
| elevation | 0.01 | 1 | -0.56 | 0.28 | 0.53 | 0.35 | 0.42 | 0.18 | 0.22 |
| human footprint index | -0.01 | -0.55 | 1 | -0.40 | -0.32 | -0.43 | -0.28 | -0.21 | -0.14 |
| landtype | 0.03 | 0.29 | -0.40 | 1 | 0.13 | 0.62 | 0.16 | 0.27 | 0.11 |
| distance to residential areas | 0.0001 | 0.53 | -0.32 | 0.14 | 1 | 0.12 | 0.48 | 0.11 | 0.17 |
| ndvi | -0.07 | 0.35 | -0.44 | 0.62 | 0.12 | 1 | 0.15 | 0.23 | 0.12 |
| distance to roads | -0.002 | 0.42 | -0.28 | 0.16 | 0.48 | 0.15 | 1 | 0.12 | 0.21 |
| slope | 0.01 | 0.18 | -0.22 | 0.27 | 0.11 | 0.23 | 0.12 | 1 | 0.07 |
| distance to water | -0.01 | 0.22 | -0.14 | 0.11 | 0.17 | 0.12 | 0.21 | 0.07 | 1 |

Table S2 Contribution percentage of the environment variables to the MaxEnt model

| **Variable** | **Percent contribution** | | |
| --- | --- | --- | --- |
|  | 2020 | 2030 scenario | |
|  |  | nature development | national park |
| elevation | 45.7 | 43.1 | 45.4 |
| human footprint index | 40.6 | 40.3 | 40.9 |
| NDVI | 5.7 | 5.8 | 3.1 |
| distance to residential areas | 2.4 | 3.8 | 3.0 |
| distance to roads | 2.2 | 2.6 | 2.6 |
| distance to water sources | 1.1 | 1.7 | 0.9 |
| aspect | 1.0 | 0.9 | 1.0 |
| slope | 0.7 | 0.9 | 0.6 |
| landtype | 0.6 | 0.8 | 2.6 |

Table S3 Summary of Selected Curve Types in Space-Time Cube

| **Types of Curves** | **Number of Locations** | **Location Percentage** |
| --- | --- | --- |
| Linear Function | 17393 | 31.71 |
| Parabola | 19226 | 35.05 |
| Exponential | 4588 | 8.36 |
| S-shaped (Gompertz) | 13139 | 23.95 |
| Mean | 506 | 0.92 |

Table S4 Positional Accuracy Summary of Curve-Fitting-Predicted 2030 Layer

| **Type** | **Minimum** | **Maximum** | **Mean** | **Median** | **Standard Deviation** |
| --- | --- | --- | --- | --- | --- |
| Prediction RMSE | 0 | 4.03 | 0.42 | 0.19 | 0.5 |
| Validation RMSE | 0 | 16.88 | 0.99 | 0.11 | 1.83 |
